# Comparison of the SF-6D and the EQ-5D in patients with coronary heart disease

**DOI:** 10.1186/1477-7525-4-20

**Published:** 2006-03-25

**Authors:** Henk F van Stel, Erik Buskens

**Affiliations:** 1 Julius Center for Health Sciences and Primary Care, University Medical Center Utrecht, room STR 6.131, PO box 85500, 3584 GA, Utrecht, The Netherlands

## Abstract

**Background:**

The SF-6D was derived from the SF-36. A single summary score is obtained allegedly preserving the descriptive richness and sensitivity to change of the SF-36 into utility measurement. We compared the SF-6D and EQ-5D on domain content, scoring distribution, pre-treatment and change scores.

**Methods:**

The SF-6D and the EQ-5D were completed prior to intervention and 1, 3, 6 and 12 months post-intervention in a study enrolling 561 patients with symptomatic coronary stenosis. Patients were randomized to off-pump coronary artery bypass surgery (CABG), standard on-pump CABG, or percutaneous transluminal coronary angioplasty (PTCA). Baseline and change over time scores were compared using parametric and non-parametric tests.

**Results:**

The relative contribution of similar domains measuring daily functioning to the utility scores differed substantially. SF-6D focused more on social functioning, while EQ-5D gave more weight to physical functioning. Pain and mental health had similar contributions. The scoring range of the EQ-5D was twice the range of the SF-6D. Before treatment, EQ-5D and SF-6D mean scores appeared similar (0.64 versus 0.63, p = 0.09). Median scores, however, differed substantially (0.69 versus 0.60), a difference exceeding the minimal important difference of both instruments. Agreement was low, with an intra-class correlation of 0.45.

Finally, we found large differences in measuring change over time. The SF-6D recorded greater intra-subject change in the PTCA-group. Only the EQ-5D recorded significant change in the CABG-groups. In the latter groups changes in SF-6D domains cancelled each other out.

**Conclusion:**

Although both instruments appear to measure similar constructs, the EQ-5D and SF-6D are quite different. The low agreement and the differences in median values, scoring range and sensitivity to change after intervention show that the EQ-5D and SF-6D yield incomparable scores in patients with coronary heart disease.

## Background

Measurement of health utility is an important part of cost-effectiveness analysis in health care. Health utility can be measured by several preference-based utility measures, of which the EuroQol (EQ-5D) [1,2] and the Health Utility Index [3] are the most widely used. Recently, a new index score, called the SF-6D, has been developed [4]. This instrument produces a summary score based on an algorithm using a subset of 11 questions from the SF-36 health status measure [5]. The major reason for developing the SF-6D was to enlarge the basis for economic evaluations, while retaining the descriptive richness and sensitivity to change of the SF-36 [6]. This reasoning is based on observations that the EQ-5D has poorer descriptive ability and is less sensitive to change compared to individual SF-36 domains [7-10]. These potential advantages of the SF-6D over alternative instruments should be substantiated in additional studies. A further point of interest may be the difference in methodology applied in deriving a utility score, which could imply that utilities with different "meaning" are obtained, thus resulting in confusion when interpreting results from studies using different instruments [11]. Potentially, policy decisions could be compromised by using utilities that are not equivalent. Therefore, we sought to assess the equivalency of the SF-6D and the EQ-5D cross-sectionally, in domain content, in scoring distribution, and in the amount of change measured after intervention. We addressed these questions by comparing the SF-6D and EQ-5D qualitatively and quantitatively, using data from two randomised controlled trials of patients with symptomatic coronary stenosis.

## Methods

We included patients with symptomatic coronary stenosis enrolled in two multicenter randomized controlled trials assessing the efficacy of the Octopus tissue stabiliser for bypass grafting. The first trial ("OctoPump") compared standard on-pump coronary artery bypass grafting (CABG) to off-pump CABG using the Octopus device with in 281 patients requiring coronary revascularisation [12,13]. The second trial ("OctoStent") compared off-pump CABG with percutaneous transluminal coronary angioplasty (PTCA) in 280 patients [14,15]. The study protocols of both trials required completion of both the EQ-5D and the SF-36 pre- and 1 month post-intervention, with follow-up until 1 year post-intervention [14]. Patients were enrolled from March 1998 to August 2000. There were no baseline differences in health status scores between the treatment arms within each trial.

### Instruments

The EQ-5D health status instrument comprises 5 questions – each with 3 levels – representing 5 health domains: pain, mood, mobility, self care and daily activities [1,2]. This results in 243 health states. Valuation was done with time-trade off, using dead as the lower anchor. The EQ-5D utility score was computed using the MVH-A1 algorithm by Dolan [16]. This algorithm yields a range from -0.594 to +1. The SF-6D uses 11 questions from the SF-36 health status measure (version 1), divided over 6 health domains: pain (6 levels), mental health (5), physical functioning (6), social functioning (5), role limitations (4) and vitality (5). The SF-6D has 18,000 health states. The valuation task for the SF-6D used the worst possible health state ('pits') on the SF-6D as is the worst outcome, valued with the standard gamble method. The SF-6D was computed using the algorithm provided by Brazier and colleagues [4]. The scoring range of the SF-6D covers +0.291 to +1. On both instruments, 1 represents full health. Both algorithms include an interaction term to account for an additional disutility in case one of the domains is scored at its most severe level.

### Statistical analysis

A qualitative assessment was carried out by comparing (dis-)similarities among domains [17] and their relative contribution to the utility scores. Relative contribution was computed as the maximal decrease of a domain divided by the total decrease in utility score for that instrument (excluding the decrease of the interaction terms). We then computed change-scores (post-intervention minus pre-intervention scores) and determined the number of missing baseline and change scores. Normality of distributions was tested with Shapiro-Wilk's *W *test [18]. The ceiling effect of each domain was assessed by computing the percentage of patients reporting no problems. To reduce the number of missing scores in the SF-6D, we imputed missing items in the SF-36 from the SF-36 domain scores. This was done by computing the mean value for a SF-36 domain, imputing that value for missing items in that domain, rounding imputed values to the nearest integer, and then recalculating the SF-6D. We performed parametric and non-parametric testing of baseline (Kruskal Wallis ANOVA) and change differences (paired t-test with 95% confidence intervals and Wilcoxon matched pairs test) between EQ-5D and SF-6D and their domains. Construct validity was assessed by computing Spearman correlations between the utility scores and between the domains of both instruments. Agreement was assessed by the Bland-Altman plot [19] and by computing an intra-class correlation coefficient (ICC). Statistical analyses were done with Statistica version 5.5 (Statsoft, 1999) and SPSS version 10.1 (SPSS Inc, 2000).

## Results

The combined study group of 561 patients consisted of mostly males (70.4%); the mean age was 60.2 years (sd 9.3).

### Domain comparison

**Figure 1:**
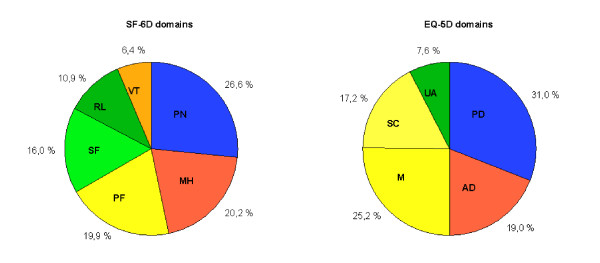
**Comparison of maxim al theoretical contribution to the utility score**. Domains measuring the same area of health have similar colors. EQ-5D dimensions: M: mobility; SC: self care; UA: usual activities; PD: pain/discomfort; AD: anxiety/depression. SF-6D dimensions: PF: physical functioning; RL: role limitation; SF: social functioning; PN: pain; MH: mental health; VT: vitality.

The EQ-5D and the SF-6D both include pain and mental health (anxiety and depression) with rather similar contributions to the overall utility scores (figure 1). Together these two domains account for about 50% in both utility scores. The other domains have less overlap. Physical functioning from the SF-6D addresses similar issues as mobility and self-care from the EQ-5D, but contributes only half as much to the SF-6D utility as mobility and self-care to the EQ-5D. The reverse is found for daily activities, which has only a limited contribution to the EQ-5D utility, while the corresponding domains from the SF-6D (social functioning and role limitations) contribute 26.9% to the utility score. The SF-6D vitality domain has no direct counterpart in the EQ-5D.

### Baseline and change scores

The SF-6D had a higher percentage of missing data, both at baseline and post-intervention (Table 1). 33 patients (5.9%) were lost to follow-up or failed to come for the post-intervention visit. Another 4.1% of the missing post-intervention utility scores, and 4.6% of the baseline scores, resulted from patients who did not fill in their questionnaires. The remainder of the missing scores resulted from individual missing items on the questionnaires. After imputation of the missing SF-36 items from SF-36 domain scores, the percentage of missing scores in the SF-6D due to missing items was reduced by half, both at baseline and post-intervention (Table 1). The median SF-6D score with imputed values did not differ from the median score without imputed values. Thus, all of the following results are based on the imputed SF-6D. There were no differences at baseline between the patients with or without a missing utility score post-intervention, so we assumed these missing scores to be at random.

**Table 1:** Percentage of missing scores

**Missing scores (%)**	**baseline**		**post-intervention**	
	total	due to missing items	total	due to missing items
EQ-5D	9.1	4.5	15.9	5.9
SF-6D	16.8	12.1	22.6	12.6
SF-6D-imputed *	10.9	6.2	16.8	6.8

*after imputation of missing SF-36 item-scores from subscale-scores

**Figure 2:**
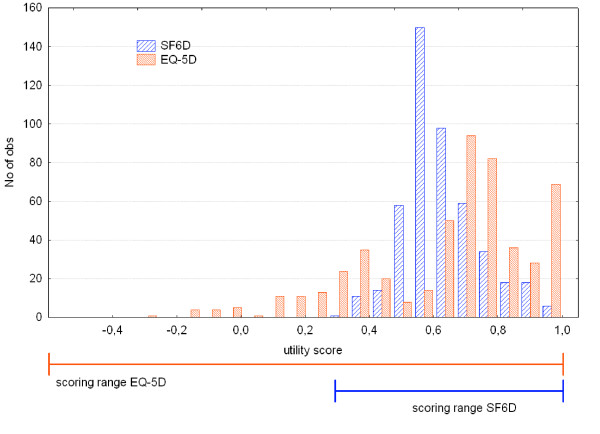
Histogram of baseline EQ-5D and SF-6D scores.

Baseline and change scores from both measures were not normally distributed (all p < 0.001). The ceiling effect in the EQ-5D domains and utility score were much larger than in the SF-6D (Table 2). There were no floor-effects in the utility scores, with minimum values of -0.32 for the EQ-5D and +0.32 for the SF-6D. The baseline EQ-5D was skewed towards perfect health, while the SF-6D was centred around 0.6 (Figure 2). The (arithmetic) mean baseline scores from the EQ-5D and SF-6D did not differ significantly using a parametric t-test: 0.64 versus 0.62 (mean difference 0.016, 95%CI 0.003 – 0.036, p = 0.09). The median values however, showed a large difference: 0.69 for EQ-5D versus 0.60 for SF-6D. Non-parametric comparison of the distributions showed highly significant differences (p < 0.001). The median baseline values have different locations in their respective scoring ranges: the median EQ-5D score was located in the top quarter, the median SF-6D in the middle part (Figure 2).

**Table 2:** Domain comparison

EQ-5D	ceiling effect at baseline (%)^#^	p-value for change in PTCA*	p-value for change in off- pump CABG*^¶^	SF-6D	ceiling effect at baseline (%)^#^	p-value for change in PTCA*	p-value for change in off- pump CABG*^¶^
pain	31.8	0.0003	0.2	pain	13.0	<0.0001	0.08
anxiety/depression	60.0	0.009	0.03	mental health	8.9	0.0008	0.02
mobility	55.1	0.004	0.0005	physical functioning	2.3	<0.0001	0.3
self care	93.1	0.3	0.3	social functioning	20.1	<0.0001	0.046
usual activities	30.5	<0.0001	0.4	role limitations	14.1	<0.0001	0. 02
				vitality	2.7	0.0005	0.6
EQ-5D	13.5	<0.0001	0.035	SF-6D	0.4	<0.0001	0.22

^#^percent maximal scores at baseline.

*wilcoxon matched pairs test between pre- and post-intervention, minimal N: EQ-5D-domains 124, SF-6D-domains 115.

^¶^change in the OctoStent off-pump CABG group; changes in the Octopump groups are comparable to this group and therefore omitted from the table

**Figure 3:**
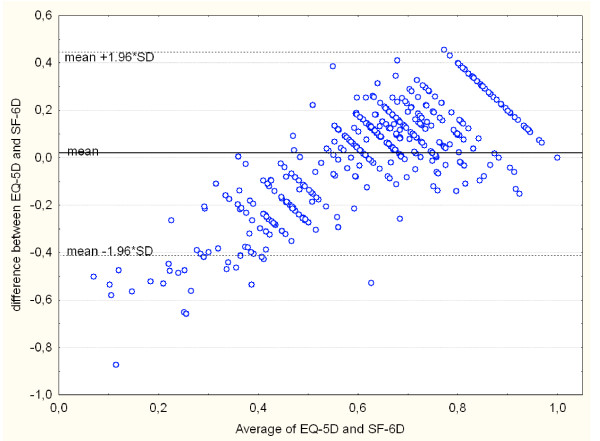
Bland-Altman plot of EQ-5D and SF-6D.

Agreement between both measures was poor, with an ICC of 0.45. The Bland-Altman plot showed proportional error, and wide limits of agreement (Figure 3). The correlation structure between the domains is rather diffuse: there are no strong correlations (>0.5), and only a few moderate correlations (Table 3). Furthermore, one would expect that domains such as physical functioning and pain (SF-6D) have the strongest correlation with their corresponding EQ5D-domains: mobility and pain. This was not the case, as both SF-6D-domains are most strongly correlated to usual activities, a domain that in it's turn has about equally strong relationships with 5 out of 6 SF-6D-domains. Only mood and mental health behave as expected, as they have a strong relationship with each other and lower correlations with all other domains.

**Table 3:** Correlation between utility domains at baseline

	**EQ-5D**				
**SF-6D**	**M**	**SC**	**UA**	**PD**	**AD**
PF	**0.31**	0.34	**0.42**	0.24	0.11
RL	0.19	0.09	**0.35**	0.30	0.21
SF	0.26	0.20	**0.41**	0.36	0.34
PN	0.32	0.23	0.48	**0.43**	0.19
MH	0.04	0.09	0.09	0.17	**0.47**
VT	0.20	0.15	0.27	0.26	0.27

All correlations (spearman) above 0.1 are significant at the p < 0.01 level; all correlations above 0.2 are significant at the p < 0.0001 level. Correlations between like domains are indicated in bold.

EQ-5D dimensions: M: mobility; SC: self care; UA: usual activities; PD: pain/discomfort; AD: anxiety/depression. SF-6D dimensions: PF: physical functioning; RL: role limitation; SF: social functioning; PN: pain; MH: mental health; VT: vitality.

**Figure 4:**
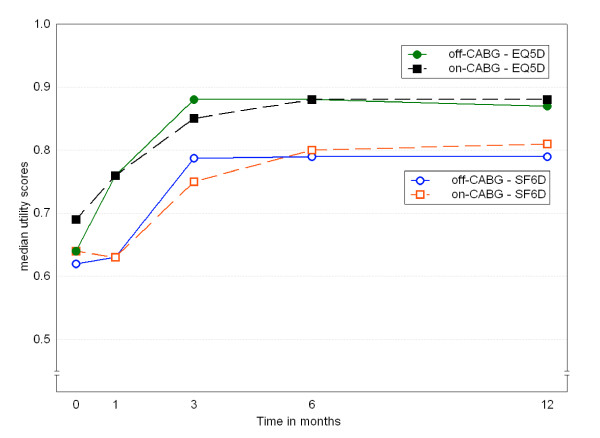
**Long term change in median utility scores**. Off-CABG = off-pump coronary artery bypass surgery; on-CABG = on-pump.

The EQ-5D and SF-6D both detected change over time in the PTCA group (Table 4). All domains from both measures, except self-care, contributed to this change (Table 2). The EQ-5D, but not the SF-6D, detected change over time in the other three groups. This lack of change in the SF-6D is partly caused by domains that change in opposite directions: significant improvement in one domain, such as mental health, is cancelled out by deterioration in other domains, such as social functioning and role limitations. The difference between EQ-5D and SF-6D in picking up change is shown in figure 4: the SF-6D lags behind, and that difference remains. The mean difference is 0.055 (95%CI 0.028 – 0.080, p < 0.0001).

**Table 4:** Comparison of baseline and post-intervention SF-6D and EQ-5D scores

		**SF-6D**		**EQ-5D**	
		baseline *	post-intervention	baseline	post-intervention
OctoStent	PTCA (n ≥ 121)	0.59 (0.10)	0.72 (0.19)^#^	0.71 (0.36)	0.80 (0.31)^#^
	Off-pump CABG (n ≥ 116)	0.61 (0.14)	0.62 (0.12)	0.69 (0.32)	0.76 (0.15)^¶^
OctoPump	Off-pump CABG (n ≥ 110)	0.62 (0.13)	0.63 (0.16)	0.69 (0.38)	0.76 (0.37)^¶^
	On-pump CABG (n ≥ 98)	0.64 (0.17)	0.63 (0.17)	0.69 (0.33)	0.76 (0.12)^¶^

data are expressed as median (interquartile range). PTCA = percutaneous transluminal coronary angioplasty; CABG = coronary artery bypass surgery

*Kruskal-Wallis anova: p = 0.016. PTCA group SF-6D scores differ from all other groups, all p ≤ 0.03; other groups do not differ from each other.

^#^significant improvement from baseline, p < 0.0001

^¶^significant improvement from baseline, p < 0.05

## Discussion

We compared the measurement properties of the EQ-5D and the SF-6D in a group of patients undergoing coronary revascularisation. We found clear differences between these utility measures: conceptual, in baseline scores and in sensitivity to change. First of all, the number of domains differs: 5 versus 6. However, the contribution of the SF-6D vitality domain, which has no counterpart in the EQ-5D, is small. Therefore, one could expect that domains tapping similar areas of health have somewhat equal contributions to the total score. This is the case for the domains pain and mood/mental health. However, the content and weights of the other domains show considerable differences, with the EQ-5D giving more weight to physical functioning and the SF-6D to social functioning. A second difference is that the recall period of both instruments is different: today for EQ-5D, versus the last four weeks (or one week in the acute version of the SF-36) for the SF-6D. The third difference is that the scoring range of the EQ-5D is twice that of the SF-6D. The location of the baseline median scores in the scoring range was quite different: in the top quarter for EQ-5D, halfway for the SF-6D. A fourth difference was that the distributions were significantly different from each other, although the mean values appeared to be similar. The difference between the median values and the limits of agreement in the Bland-Altman plot exceed the minimal clinically important difference of both SF-6D and EQ-5D [20,21]. The lack of agreement is further exemplified by the low ICC.

A fifth difference is found in the sensitivity to change. Both measures recorded change in the PTCA group, but differed in the CABG groups: EQ-5D scores improved significantly, but SF-6D scores did not change. The SF-6D recorded greater change than the EQ-5D in the PTCA group, despite its narrower scoring range. In the CABG groups, the change in the EQ-5D was caused by change in anxiety/depression and mobility. There was however no corresponding improvement in the SF-6D physical functioning domain. The significant deterioration in social functioning and role limitations cancelled out the improvement in mental health, resulting in no change in the overall SF-6D score. Another important reason for the difference in amount of change after CABG may lie in the differing recall periods: with a post-intervention assessment at one month, the 4-week recall period of the SF-36 encompasses both the intervention and recovery period, as compared to today's health status in the EQ-5D. However, the difference between SF-6D and EQ-5D remains at the subsequent measurements. This cannot be fully explained by different recall periods, as patients are stable by 6 months, and today's health should not differ that much from that over the last 4 weeks.

Both measures display non-normal distributions, both at baseline and in change over time. The EQ-5D is skewed towards good health, which creates a ceiling effect. The SF-6D is highly centred on the middle of the scoring range (see figure 1). The difference in scoring range may be explained by differences in reference state for the valuation task and the valuation technique. Two-thirds of the respondents valued the worst possible health state health state of the SF-6D as better than dead, causing the lower limit of the SF-6D to be quite a bit higher than zero [4]. The EQ-5D valuation study used dead as the lower anchor, resulting in negative scores for the worst health states [16]. The valuation studies of both instruments used different valuation techniques. The standard gamble method, used for the SF-6D, generally gives somewhat higher valuations than time-trade off (used for MVH-A1 tariff) [22,23], but these differences are not large enough to explain the narrower scoring range of the SF-6D. The difference in scoring range implies that apparently similar baseline scores and change scores are not equivalent, prohibiting direct comparisons between utility scores obtained by different instruments. More detailed discussions of the differences in valuation methods and scoring algorithms are given by Brazier and coworkers [24] and Bryan and Longworth [25].

A substantial part of the missing SF-6D scores were caused by incompletely filled-in questionnaires. The algorithm of the SF-6D requires that all relevant questions are answered. However, the algorithm of the domain scores of the SF-36 allows a certain amount of missing scores, which are imputed with the mean value of the completed items of that domain [5]. We used that technique to reduce the number of missing scores in the SF-6D; imputing a value for missing items in a SF-36 domain using the mean value for that domain. This way, the amount of missing scores in the SF-6D due to incomplete questionnaires was halved, from about 12% to 6% of the total number of SF-6D scores. Imputation did not affect the median values. Note however that this solution would not be viable if the SF-6D would be administered without the other SF-36 questions.

Recently, some studies were done that compared the EQ-5D and the SF-6D, as in our study [21,24-29]. In a study comparing seven patient groups, Brazier and coworkers found overall similar mean scores for the two measures in patients with mild diseases [24], but baseline values clearly differ in more severe patients such as liver transplant patients [26] and patients with a recent stroke [21]. These studies confirmed some of the disagreements we found: differing descriptive content and differing scoring range [24,26,29]. The pattern of correlations between domains we found was similar to the Brazier study, except that the magnitude of the correlations was much lower. Despite the strong correlation between the utility scores, these data do not support the construct validity, as the correlation structure was rather diffuse with only moderate correlations. Only mood/mental health behaved as expected (i.e. a strong correlation with each other, and low correlations with other domains).

The sensitivity to change of the SF-6D remains unclear: Pickard and colleagues found that the SF-6D was as sensitive as the EQ-5D in stroke patients – although the SF-6D also changed in patients who reported themselves as unchanged [21]. Other studies, including ours, found no change in SF-6D after intervention, compared to significant changes in the EQ-5D [26,29].

These differences at baseline and in change over time imply that changes in utility and/or quality adjusted life years based on different instruments cannot be directly compared. Furthermore, these differences are larger than the minimal clinically important difference, which will influence conclusions of cost-effectiveness analysis and clinical decision-making.

## Conclusion

In conclusion, the EQ-5D and SF-6D are not equivalent, despite some resemblances. Although the mean utility scores appear to be similar, the differences in median values, scoring range and sensitivity to change after intervention and the low agreement show that the EQ-5D and SF-6D yield incomparable scores. Even within a group of patients with the same diagnosis, the EQ-5D and SF-6D yield different scores, while sensitivity to change seems to be influenced by the type of intervention. The SF-6D has better distributional properties than the EQ-5D, but that did not result in improved sensitivity to change. However, it cannot be said which instrument is correct. Clearly, the SF-6D measures something else than the EQ-5D, and these instruments cannot be used interchangeably.

Currently, there is no clear benefit in using the SF-6D in clinical studies instead of the EQ-5D, as the SF-6D is not clearly better. As the EQ-5D presently is generally accepted, it may be preferred, thus obtaining results comparable with previous studies.

## Authors' contributions

HFvS participated in the design of the study, performed the statistical analysis and drafted the manuscript. EB conceived of the study and participated in it's design. Both authors read and approved the final manuscript.
